# On the bias of complete‐ and shifting‐case meta‐regressions with missing covariates

**DOI:** 10.1002/jrsm.1558

**Published:** 2022-04-07

**Authors:** Jacob M. Schauer, Jihyun Lee, Karina Diaz, Therese D. Pigott

**Affiliations:** ^1^ Department of Preventive Medicine Northwestern University Chicago Illinois USA; ^2^ Department of Educational Psychology University of Texas at Austin Austin Texas USA; ^3^ Consortium for Policy Research in Education University of Pennsylvania Philadelphia Pennsylvania USA; ^4^ College of Education & Human Development Georgia State University Atlanta Georgia USA

**Keywords:** complete‐case analysis, meta‐regression, missing data, shifting units of analysis

## Abstract

Missing covariates is a common issue when fitting meta‐regression models. Standard practice for handling missing covariates tends to involve one of two approaches. In a complete‐case analysis, effect sizes for which relevant covariates are missing are omitted from model estimation. Alternatively, researchers have employed the so‐called "shifting units of analysis" wherein complete‐case analyses are conducted on only certain subsets of relevant covariates. In this article, we clarify conditions under which these approaches generate unbiased estimates of regression coefficients. We find that unbiased estimates are possible when the probability of observing a covariate is completely independent of effect sizes. When that does not hold, regression coefficient estimates may be biased. We study the potential magnitude of that bias assuming a log‐linear model of missingness and find that the bias can be substantial, as large as Cohen's *d* = 0.4–0.8 depending on the missingness mechanism.

**Highlights**

Missing covariates are a common problem when conducting meta‐regressions. A common practice for meta‐regression analyses has been to ignore effects for which covariates are missing. However, a vast statistical literature suggests that analyses that ignore missing data can only provide accurate estimates of relevant quantitites under certain conditions. In this article, we examine conditions under which ignoring missing covariates in a meta‐regression can still lead to unbiased estimation of regression coefficients. We also investigate the possible magnitude and sources of bias when those conditions do not hold. Our findings highlight that substantial bias can be induced by ignoring missing data in a meta‐regression.

## 1 INTRODUCTION

Meta‐regression is a useful tool for studying important sources of variation between effects in a meta‐analysis.^1^, ^2^ Analyses of these models in the absence of missing data have been studied thoroughly in the literature.^3^, ^4^, ^5^, ^6^, ^7^ However, it is common for meta‐analytic datasets to be missing data.^8^ In the context of meta‐regression, issues with missing data frequently involve missing covariates.^9^, ^10^

Precisely how to proceed with a meta‐regression when missing covariates remains something of an open question. Statistical guidance suggests that analyses ought to consider the mechanism that causes covariates to be missing.^9^, ^11^ However, it appears that doing so is less common in practice for meta‐analyses. A recent review found that meta‐regressions with missing data tend to take one of two strategies.^10^ An analyst may conduct a complete‐case analysis (CCA) that excludes any effects for which a relevant covariate is missing (i.e., only analyze complete cases). This is often referred to as “listwise deletion” in data analyses. However, if there are very few such effects, a common approach is to use shifting units of analysis, which we refer to in this article as a shifting‐case analysis (SCA).^12^ In an SCA, analysts fit a series of meta‐regression models on subsets of relevant covariates, so that each model selectively omits certain covariates. This is equivalent to “pairwise deletion” in data analyses.

Both CCA and SCA ignore effects for which a covariate is missing. Ignoring missing data can potentially lead to biased estimates of parameters of interest.^13^, ^14^ Despite authors pointing out such issues in meta‐analysis, these methods continue to enjoy widespread use.^11^ Existing meta‐analysis literature on this discussion has yet to detail precisely how much bias can arise in a complete‐ or shifting‐case analysis, nor is there exhaustive guidance on when these methods produce unbiased estimates. In short, there is an understanding that these methods can induce bias, but less is known about how much and under what conditions.

This article examines the potential bias of complete‐ and shifting‐case analyses. The following section provides a demonstration of these methods on data concerning a meta‐analysis of substance abuse interventions.^15^ We then introduce a statistical framework for studying bias for incomplete data meta‐regressions that incorporates a model for whether or not a covariate is observed. Using this framework, we describe conditions under which CCA and SCA are unbiased. When these conditions are not met, we derive an approximation for the bias of CCA and SCA using standard models for missingness and examine the magnitude of bias. We find that bias is highly dependent on the precise mechanism by which data are missing, and is less reliant on more traditional missingness mechanism classifications (e.g., missing at random vs. not at random).

## 2 EXAMPLE: SUBSTANCE ABUSE INTERVENTIONS

Tanner‐Smith et al.^15^ conducted a meta‐analysis that examined the effects of substance abuse interventions on future substance use among adolescents. The studies included in this meta‐analysis involved a variety of different treatment types (e.g., cognitive behavioral therapy, family therapy, and pharmacological therapy) and treatment intensities (measured in hours per week), and were carried out in a variety of contexts, including in‐patient and out‐patient centers. Tanner‐Smith et al. used meta‐regression models to study potential moderators of these effects, and their analyses had to contend with a number of effects that were missing covariates. While in practice, models were estimated via the expectation–maximization (EM) algorithm rather than complete‐ or shifting‐case methods, we use a subset of this data in order to illustrate complete‐ and shifting‐case analyses.

Consider a subset of the Tanner‐Smith et al. data comprising 74 effect estimates of substance abuse interventions from 46 studies. These effect estimates involve contrasts between groups in a study that are subjected to different treatment conditions, denoted in the data as Group 1 and Group 2, so that each treatment effect can be thought of as Group 1 minus Group 2. Typically, researchers avoided no‐treatment or placebo conditions in studies over ethical concerns surrounding the failure to treat adolescents with substance abuse disorders. Thus, contrasts within studies (i.e., effect estimates) tended to focus on a specific treatment of interest to the researcher versus some alternate treatment. Effect estimates are reported on the scale of bias‐corrected standardized mean differences.

Suppose the analysis of interest involves the impact of high‐ versus low‐intensity interventions on treatment effects, where a high‐intensity intervention consisted of more than 1.5 h per week of treatment. Then this analysis might use a pair of binary covariates for each effect: one would indicate whether Group 1 received a high‐intensity intervention (i.e., *X*_1_ = 1 if Group 1 treatment was high‐intensity) and the other would indicate whether Group 2 received a high intensity (i.e., *X*_2_ = 1 if Group 2 treatment was high‐intensity). The relevant meta‐regression model would regress the effect estimates on these two covariates.

In the data, the treatment intensity is missing for some of the effects, and Table 1 summarizes missingness for these covariates. Table 1 shows that only 37 of the 74 (50%) have a reported treatment intensity for both groups (i.e., *X*_1_ and *X*_2_ are both observed), but that 54 (73%) of effects report Group 1's treatment intensity (i.e., *X*_1_ is observed) and 41 (55%) effects report Group 2's treatment intensity.

**TABLE 1:** The total number and percentage of effect sizes that are missing covariates regarding whether Group 1 or Group 2 received high‐intensity interventions in the substance abuse intervention meta‐analysis

Group 1 Hi‐intensity	Group 2 Hi‐intensity	Count	Percent
Observed	Observed	37	0.50
Observed	Missing	17	0.23
Missing	Observed	4	0.05
Missing	Missing	16	0.22

A complete‐case analysis would include only the 37 effects for which both covariates were observed. Using robust variance estimation to account for dependence between effect sizes, a CCA would result in the coefficient estimates and standard errors displayed in the first column of Table 2. Based on these estimates, when Group 1 receives a high‐intensity treatment, we would expect an effect to be larger by *d* = 0.44 (in standard deviation units) than when Group 1 receives a low‐intensity treatment, which is statistically significant at the *α* = 0.10 level. Note that the estimated between‐effect variance is ${\hat{\tau }}^{2}=0.08$.

**TABLE 2:** The meta‐regression results for the model regressing effect sizes on high‐intensity indicator variables when using complete‐ and shifting‐case analyses

Term	Complete‐case	Shifting‐case Group 1	Shifting‐case Group 2
Intercept	0.11 (SE = 0.06, *p* = 0.11)	0.14 (SE = 0.06, *p* = 0.02)	0.15 (SE = 0.06, *p* = 0.03)
Group 1 Hi‐Int.	0.44 (SE = 0.16, *p* = 0.06)	0.27 (SE = 0.15, *p* = 0.07)	–
Group 2 Hi‐Int.	−0.21 (SE = 0.26, *p* = 0.46)	–	0.16 (SE = 0.26, *p* = 0.54)
Variance comp. *τ* ^2^	0.08	0.06	0.09

However, the model above is estimated on only half of the data. Concern over using a small proportion of the data, or a relatively few number of effects often leads meta‐analysts to opt for a shifting‐case analysis. An example of an SCA would use the 54 effects for which Group 1's treatment intensity is observed (i.e., *X*_1_ is observed), but only including *X*_1_ in the model. Doing so leads to the estimates in second column of Table 2. Note that the coefficient estimate for Group 1's treatment intensity is still positive, but is roughly 60% the magnitude of the estimate in the complete‐case model.

Finally, an analogous model in an SCA would include the 41 effects for which Group 2's intensity is observed, and include only that covariate in the model. The third column of Table 2 shows that this results in a coefficient estimate for Group 2's treatment intensity (0.16) that is in the opposite direction of the estimate from the CCA (−0.21).

It should be noted that all of these estimates and comparisons between them ought to be interpreted with caution. The complete‐case analysis includes only half of the effect sizes, which comprises a missingness rate well beyond what might be considered negligible.^16^, ^17^ The shifting‐case analyses include more of the data, but because each shifting‐case model omits one of the covariates, these models are not equivalent to the model that includes both covariates.^18^ It could even be argued that the parameters in the model with both covariates are not comparable to parameters in models with only one covariate; coefficients in a model with multiple covariates must be interpreted in relation to other variables in the model. The remainder of this article quantifies the bias induced by omitting effect sizes and/or covariates from meta‐regressions.

## 3 MODEL AND NOTATION

Suppose a meta‐analysis involves *k* effects estimated from collection of studies. For the *i*th effect, let *T*_*i*_ be the estimate of the effect parameter *θ*_*i*_, and let *v*_*i*_ be the estimation error variance of *T*_*i*_. Denote a vector of covariates that pertain to effect estimate *T*_*i*_ as *X*_*i*_ = [1, *X*_*i*1_, …, *X*_*ip*_]. Note that the first element of *X*_*i*_ is a 1, which corresponds to an intercept term in a meta‐regression model, and that *X*_*ij*_ for *j* = 1, …*p* corresponds to different covariates. The meta‐regression model can be expressed as:

(1) $$T_{i}|X_{i},v_{i},\eta =X_{i}\beta +u_{i}+e_{i}.$$

Here, $\beta \in {\mathbb{R}}^{p+1}$ is the vector of regression coefficients. The estimation errors *e*_*i*_ are typically assumed to be normally distributed with mean zero and variance *V* [*e*_*i*_] = *v*_*i*_. This assumption is true of some effect size indices and is a very accurate large‐sample approximation for others.^19^ The term *u*_*i*_ represents the random effect such that *u*_*i*_ ⊥ *e*_*i*_ and *V* [*u*_*i*_] = *τ* ^2^. This model is equivalent to the standard mixed‐effects meta‐regression model, and it is also consistent with subgroup analysis models.^19^, ^20^ The vector *η* = [*β*, *τ* ^2^] refers to the parameters of model. Under a fixed‐effects model, it is assumed that *τ* ^2^ = 0, in which case *η* = *β*, and *u*_*i*_ ≡ 0.

A common assumption in random effects meta‐regression is that the random effects *u*_*i*_ are independent and normally distributed with mean zero and variance *τ* ^2^:^20^, ^21^, ^22^, ^23^

$$u_{i}∼N\left(0,\tau^{2}\right).$$

This could correspond to a scenario of *k* independent effect estimates presumably from *k* different studies. In that case, the distribution *p*(*T*|*X*, *v*, *η*) can be written as

(2) $$p\left(T_{i}|X_{i},v_{i},\eta \right)=\frac{1}{\sqrt{2\pi \left(\tau^{2}+v_{i}\right)}}e^{-\frac{{\left(T_{i}-X_{i}\beta \right)}^{2}}{2\left(\tau^{2}+v_{i}\right)}}.$$

Thus, the joint likelihood for all *k* effects can be written as:

(3) $$p\left(T|X,v,\eta \right)={\left(2\pi \right)}^{-k/2}\left[\prod_{i=1}^{k}\left(\tau^{2}+v_{i}\right)\right]e^{-\sum_{i=1}^{k}\frac{{\left(T_{i}-X_{i}\beta \right)}^{2}}{2\left(\tau^{2}+v_{i}\right)}},$$

where $T\in {\mathbb{R}}^{k}$ is the vector of effect estimates, $v\in {\mathbb{R}}^{k}$ is the vector of estimation variances, and $X\in {\mathbb{R}}^{k\times \left(p+1\right)}$ is the matrix of covariates where each row of **X** is simply the row vector *X*_*i*_. Note that the functions in both (2) and (3) assume that all of the p covariates are observed. Equation (3) is referred to as the complete‐data likelihood function.^13^, ^24^ We note that a meta‐regression with no missing data will be accurate if the complete‐data model is correctly specified. Thus, to illustrate the properties of incomplete data meta‐regression, we assume that the complete‐data model is correctly specified.

The vector of regression coefficient estimates for the complete‐data model when there is no missing data is typically estimated by

(4) $$\hat{\beta }={\left(X^{T}\mathrm{WX}\right)}^{-1}X^{T}\mathrm{WT}.$$

Here, **W** = diag[*w*_*i*_] is the diagonal matrix of weights such that *w*_*i*_ = 1/(*v*_*i*_ + *τ* ^2^). The covariance matrix of $\hat{\beta }$ is given by

(5) $$V\left[\hat{\beta }\right]={\left(X^{T}\mathrm{WX}\right)}^{-1}$$

Note that the weights involve the true variance component *τ* ^2^. In practice, *τ* ^2^ must be estimated by ${\hat{\tau }}^{2}$, and the resulting weights used in analyses can be written ${\hat{w}}_{i}=1/\left(v_{i}+{\hat{\tau }}^{2}\right)$. For the sake of simplicity, we use *w*_*i*_ to derive results in this article, and so results do not depend on variance component estimators. Presumably, use of ${\hat{w}}_{i}$ would induce additional variation into analyses.

The substance abuse data contains multiple effect estimates per study that are likely correlated. This differs from the model above. However, we can expand this model to account for dependent effect sizes by assuming that $T_{i}\in {\mathbb{R}}^{k_{i}}$ is a vector of *k*_*i*_ effects from the same study, *e*_*i*_ is vector of estimation errors, *u*_*i*_ is a vector of random effects, and *e*_*i*_ + *u*_*i*_ has covariance matrix ∑_*i*_. In this model, *X*_*i*_ is a matrix of covariates for each effect in *T*_*i*_. The resulting formulas for the complete‐data likelihood function and coefficient estimators will be more complex (including a variance–covariance weight matrix), but they will have a similar form as the independent effect size model.

Not all relevant variables may be observed in a meta‐analytic dataset. Let *R*_*i*_ be a vector of response indicators that correspond with effect *i*. This article concerns missing covariates, and we assume that *T*_*i*_ and *v*_*i*_ are observed for every effect of interest in a meta‐analysis. Thus, each element *R*_*ij*_ of *R*_*i*_ corresponds to a covariate *X*_*ij*_. The *R*_*ij*_ take a value of either 0 or 1: *R*_*ij*_ = 1 indicates the corresponding *X*_*ij*_ is observed and *R*_*ij*_ = 0, indicates a that the corresponding *X*_*ij*_ is not observed. Note that $R_{i}\in ℛ\equiv {\left\{0,1\right\}}^{p}$ is a vector of 0 and 1 s of length *p*. For instance, *X*_*i*2_ were missing, this would be indicated by *R*_*i*2_ = 0.

Denote *O* = {(*i*, *j*): *R*_*ij*_ = 1} as the indices of covariates that are observed and *M* = {(*i*, *j*): *R*_*ij*_ = 0} be the set of indices for missing covariates. Then, the complete‐data model can be written as

(6) $$p\left(T|X,v,\eta \right)=p\left(T|X_{O},X_{M},v,\eta \right).$$

Note that the complete‐data model depends on entries of **X**_M_, which are unobserved. It is worth pointing out that the complete‐data model, which refers to the model with no missing data, is distinct from the complete‐case analysis, which is an estimation procedure that conditions only on observed data.

### 3.1 Complete‐case estimators

A common approach in meta‐regression with missing covariates is to use a complete‐case analysis.^10^, ^11^ This approach simply omits rows in the data for which any covariate is missing. Thus, this analysis method only uses effects and covariates for which *R*_*i*_ = [1, …, 1] = 𝟙.

Let *C* = {*i*: *R*_*i*_ = 𝟙} index all relevant effects *i* such that *R*_*i*_ = 𝟙, so that **X**_C_ is the matrix of covariates such *R*_*i*_ = 𝟙, **T**_C_ is the corresponding subset of effect estimates, and **W**_C_ is the corresponding subset of weights. The CCA estimates the coefficients *β* with

(7) $${\hat{\beta }}_{C}={\left(X_{C}^{T}W_{C}X_{C}\right)}^{-1}X_{C}^{T}W_{C}T_{C}.$$

### 3.2 Shifting‐case estimators

When there are multiple covariates of interest, each of which has some missingness, there may only be a few effects for which all covariates of interest are observed. When that happens, a complete‐case analysis can be unfeasible. A common solution to this in meta‐analysis is to use an available‐case analysis.^11^ In practice, an available‐case meta‐regression is often equivalent to a shifting‐case analysis, referred to in the literature as shifting units of analysis.^10^, ^12^

Shifting‐case analyses involve fitting multiple regression models, each including a subset of the covariates of interest. Sometimes this even takes the form of regressing effect estimates on one covariate at a time.^10^, ^11^ In the substance abuse data example, we focused on two covariates of interest *X*_*i*1_ and *X*_*i*2_. The SCA first regressed *T*_*i*_ on observed values of *X*_*i*1_. This regression included observations for which both *X*_*i*1_ and *X*_*i*2_ are observed (i.e., *R*_*i*_ = [1, 1]) and observations for which *X*_*i*1_ is observed but *X*_*i*2_ is missing (i.e., *R*_*i*_ = [1, 0]). We then regressed *T*_*i*_ on *X*_*i*2_, which included effects for which *R*_*i*_ ∈{[1, 1], [0, 1]}. In sum, the SCA demonstrated in the previous section involved two regressions, each of which conditioned on different sets of missingness patterns.

To formalize SCA estimators, consider a single regression in an SCA, and let *S* index the component of *X*_*i*_ (i.e., the intercept term and relevant covariates) included in that model *S* = {*j*: *j* = 0 or *X*_*ij*_ in analysis}. Let *E* be the complement of *S* so that *E* indexes the covariates excluded from the regression. Then, the regression is used to estimate and make inferences about coefficients *β*_S_. In the following section, we discuss *β*_*S*_ and its relationship to *β*, but here assume that the target of inference for an SCA is *β* and hence *β*_*S*_ comprises a subset of the components of *β*. For instance, in the first substance abuse SCA regression, *T*_*i*_ was regressed on only *X*_*i*1_, so that *β*_S_ = [*β*_0_, *β*_1_].

Denote ${ℛ}_{j}$ as the set of missingness patterns such that all included covariates are observed: ${ℛ}_{j}=\left\{R\in ℛ:R_{S}=1\right\}$. Note that ${ℛ}_{j}$ contains missingness patterns such that all the included covariates are observed, but any excluded covariates may be either observed or unobserved. For instance, in the first substance abuse SCA regression of *T*_*i*_ on *X*_*i*1_, the analysis included effects such that $R_{i}\in {ℛ}_{1}=\left\{\left[1,1\right],\left[1,0\right]\right\}$. Finally, let *U* denote the indices *i* of effects for which *X*_*iS*_ are observed; note that *U* depends on *S*, so we may write $U\left(S\right)=\left\{i:R_{i}\in {ℛ}_{j}\right\}$. Then, the shifting‐case estimators for *β*_*S*_ are given by:

(8) $${\hat{\beta }}_{S}={\left(X_{\mathrm{US}}^{T}W_{U}X_{\mathrm{US}}\right)}^{-1}X_{\mathrm{US}}^{T}W_{U}T_{U},$$

where **X**_*US*_ contains the columns (*S*) of **X** that pertain to the covariates that are included in the SCA regression, and the rows (*U*) for which all of those covariates are observed. The matrix **W**_*U*_ is a square matrix containing the relevant rows and columns of **W** for which *X*_*iS*_ are observed, while **T**_*U*_ contains the effect sizes in **T** for which *X*_*iS*_ is observed.

### 3.3 Omitted variables

A common concern in meta‐regression is that models may not be able to account for all relevant covariates, either due to sample size constraints or because some covariates were not observed.^25^ Such concerns pertain to meta‐regressions both with and without missing data. In contrast to primary data analysis, meta‐analysts tend to have little control over the availability of covariates relevant for a meta‐regression. Information regarding covariates must be extracted from primary studies and this process is restricted by the ways in which research is reported. Some studies may not clearly report covariates deemed of interest in a meta‐regression. Even for key conceptually or theoretically important moderators, meta‐regression models must often contend with variation in reporting of such moderators across studies. Thus, the issue of omitted variables in meta‐regression is both prevalent and difficult to overcome (e.g., via additional data collection).

The implication of omitting observed variables in SCA can be understood via the parameter *β*_*S*_. It has been noted that there are various conditions under which components of *β*_*S*_ are unequal to their counterparts in *β*.^26^ For instance, it can be the case that $\beta_{S}=\left[{\tilde{\beta }}_{0},{\tilde{\beta }}_{1}\right]\neq \left[\beta_{0},\beta_{1}\right]\subset \beta$. The difference between *β*_*S*_ and components of *β* is often referred to as omitted variable bias in the statistical and econometric literature.^27^, ^28^ This conception inherently assumes that *β* in the full model is of interest to the analyst, which may not necessarily be the case. Indeed, one may assume that *β*_*S*_ is of interest, rather than *β*, so that the components of *β*_S_ comprise parameters distinct from *β*. In this approach, *β*_S_ characterizes the relationship between *X*_*i*S_ and *T*_*i*_ in a more restricted model that does not account for *X*_*i*E_. This could be consistent with analyses that seek to summarize effects within specific subgroups of studies delineated by covariates.

We refer to the difference between *β*_S_ and *β* as omitted variable bias, in keeping with the literature on linear models. In doing so, we treat SCA as a missing data analytic strategy, wherein the target of inference is *β*. Subsequent sections present findings on bias induced by omitting observed covariates in an SCA, which reflect the findings of Lipsey (2003), who points out that interpretation of meta‐regression coefficients when covariates are omitted can lead to misleading interpretations about the correlates of effective interventions. However, if the intent of the analysis is to examine restricted models or specific subgroups of effects/studies, the omitted variable bias presented in this article may be less applicable, though Lipsey's caveats for interpreting such models may still apply.

### 3.4 Missingness mechanisms

Both the complete‐ and shifting‐case estimators are analyses of incomplete data. Analyses of incomplete data require some assumption about why data are missing, which is referred to as the missingness *mechanism*. The mechanism by which missingness arises is typically modeled through the distribution of *R*. Let *ψ* denote the parameter (or vector of parameters) that index the distribution of *R* so that the probability mass function of *R* can be written as *p*(*R*|*T*, *X*, *v*, *ψ*). Assumptions about the missingness mechanism are therefore equivalent to assumptions about *p*(*R*|*T*, *X*, *v*, *ψ*).

Rubin^29^ defined three types of mechanisms in terms of the distribution of *R*. Data could be missing completely at random (MCAR), which means that the probability that a given value is missing is independent of all of the observed or unobserved data:

$$p\left(R|T,X_{O},X_{M},v,\psi \right)=p\left(R|\psi \right).$$

MCAR implies that probability that a given value is missing depends only on the missingness parameter *ψ*.

Covariates could be missing at random (MAR), which implies the distribution of missingness depends only on observed data and the missingness parameter:

$$p\left(R|T,X_{O},X_{M},v,\psi \right)=p\left(R|T,X_{O},v,\psi \right).$$

MAR differs from MCAR in that missingness might be related to observed values. As an example, if studies with larger standard errors are less likely to report the racial composition of their samples, then missingness would depend on the (observed) estimation error variances. Data missing according to this mechanism would violate an assumption of MCAR, since missingness is related to an observed value.

Finally, data are said to be missing not at random (MNAR) if the distribution of *R* depends on unobserved data in some way. In the context of the meta‐regression data, this would imply that *R* is related to **X**_M_, so that the probability of a covariate not being observed depends on the value of the covariate itself. For instance, data would be MNAR if studies with larger standard errors and a greater proportion of minorities are less likely to report the racial composition of their samples because the likelihood that racial composition is not reported will depend on the composition itself.

A related concept in missing data is that of ignorability, which means that the missingness pattern does not contribute any additional information. When missing data are ignorable, it is not necessary to know (or estimate) *ψ* in order to conduct inference on *η*.^13^, ^14^, ^24^, ^30^ In practice, missing data are ignorable if they are MAR and if *ψ* and *η* are distinct.

## 4 CONDITIONAL INCOMPLETE DATA META‐REGRESSION

Because both complete‐ and available‐case analyses depend on the value of *R*_*i*_, they can be seen as models that condition on missingness. Models that condition on missingness are not necessarily identical to the complete‐data model, which is the model of interest, because the complete‐data model does not condition on *R*_*i*_. Yet, CCA and SCA proceed as if the complete‐data and conditional models on missingness are equivalent. Doing so ignores the missingness mechanism and its potential impact on the accuracy of analytic results.

The complete‐data model can be related to the conditional models through the distribution of missingness *R*_*i*_. This approach is referred to as a *selection model* in the missing data literature.^13^, ^24^, ^30^ We can write the selection model for meta‐regression with missing covariates as:

(9) $$p\left(T_{i}|X_{i},v_{i},R_{i}\in {ℛ}_{j},\eta ,\psi \right)=\frac{p\left(R_{i}\in {ℛ}_{j}|T_{i},X_{i},v_{i},\psi \right)p\left(T_{i}|X_{i},v_{i},\eta \right)}{p\left(R_{i}\in {ℛ}_{j}|X_{i},v_{i},\psi ,\eta \right)},$$

where *ψ* indexes the distribution of *R*|*T*, *X*, *v*. Here, ${ℛ}_{j}$ refers to the relevant subset of ℛ on which the analysis conditions; for a CCA, ${ℛ}_{j}=\left\{1\right\}$.

Equation (9) describes the conditional model as a function of the complete‐data model *p*(*T*_*i*_|*X*_*i*_, *v*_*i*_, *η*) and a selection model $p\left(R_{i}\in {ℛ}_{j}|T_{i},X_{i},v_{i},\psi \right)$ that gives the probability that a given set of covariates are observed. The denominator on the right hand side of (9) is a normalizing factor that is equivalent to the probability of observing the missingness pattern ${ℛ}_{j}$ given the estimation error variance *v*_*i*_ and the observed and unobserved covariates in the vector *X*_*i*_, and can be written as

(10) $$p\left(R_{i}\in {ℛ}_{j}|X_{i},v_{i},\psi ,\eta \right)=\int p\left(R_{i}\in {ℛ}_{j}|T_{i},X_{i},v_{i},\psi \right)p\left(T_{i}|X_{i},v_{i},\eta \right){\mathrm{dT}}_{i}.$$

Note that when the complete‐data model in (2) is not equivalent to the conditional model in (9), the resulting coefficient estimators in a meta‐regression can be biased. To see this, we can write:

(11) $$E\left[T_{i}|X_{i},v_{i},R_{i}\in {ℛ}_{j}\right]=E\left[T_{i}|X_{i},v_{i}\right]+\delta_{\mathrm{ij}}=X_{i}\beta +\delta_{\mathrm{ij}}.$$

Here, we see that the expectation of *T*_*i*_ given *X*_*i*_ and *R*_*i*_ can be written as the complete‐data expectation *X*_*i*_ *β* (i.e., the regression model) plus a bias term *δ*_*ij*_. The bias term *δ*_*ij*_ refers to the bias induced in the regression model due to conditioning on missingness pattern ${ℛ}_{j}$, which can affect individual components of *η*. If *δ*_*ij*_ ≠ 0, it follows that conditioning on *R*_*i*_ induces bias in the distribution of *T*_*i*_ used in an analysis. Because the CCA estimator (7) and SCA estimator in (8) are weighted averages of the *T*_*i*_, they can be biased if *δ*_*ij*_ ≠ 0. The precise magnitude of the *δ*_*ij*_ will depend on the selection model in (9) and hence on the missingness mechanism. It is worth noting that the subsequent sections show that bias depends on the precise selection model rather than the class of mechanism (MCAR or MAR).

A standard approach for modeling missingness mechanisms for covariates is to assume *R*_*i*_ follows some log‐linear distribution.^31^ Various authors have described approaches to modeling *R* for missing covariates in generalized linear models that include logistic and multinomial logistic models.^32^, ^33^, ^34^ Thus, one class of models for missingness would involve the logit probability of observing some missingness patterns $R_{i}\in {ℛ}_{j}\subset ℛ$:

(12) $$\text{logit}\left[p\left(R_{i}\in {ℛ}_{j}|T_{i},X_{i},v_{i}\right)\right]=\sum_{m=0}^{m_{j}}\psi_{\mathrm{mj}}f_{\mathrm{mj}}\left(T_{i},X_{i},v_{i}\right).$$

Here, *f*_*mj*_(*T*_*i*_, *X*_*i*_, and *v*_*i*_) are assumed to be differentiable basis functions of the data and *m*_*j*_ is the number of terms in the selection model. In theory, *m*_*j*_ could be arbitrarily large, but the model is only estimable if *m*_*j*_ < *k*. Finally, we assume *f*_0*j*_(*T*_*i*_, *X*_*i*_, *v*_*i*_) = 1, so that *ψ*_0*j*_ would be the intercept term for the logit model for the set of missingness patterns ${ℛ}_{j}$.

In general, it is impossible to know whether a selection model is correctly specified, but the formulation in (12) offers a few important advantages. First, it is fairly general: the only assumption made of the basic functions *f*_*mj*_ is that they are differentiable, which means model (12) allows for nonlinear or interaction terms. Second, it expresses the relationships between the probability of the event $\left\{R_{i}\in {ℛ}_{j}\right\}$ and observed variables on the scale of the log odds ratio, a well‐understood scale in meta‐analysis. Third, it allows for closed‐form expressions for the approximate bias of coefficient estimates by virtue of the logit link function. Thus, it comprises a large class of models for selection that can be more clearly interpreted.

### 4.1 Approximate bias for log‐linear selection models

As argued above, the bias of complete‐case estimators ${\hat{\beta }}_{C}$ or shifting‐case estimators ${\hat{\beta }}_{S}$ will depend in some way on the bias *δ*_*ij*_ induced in *T*_*i*_ by conditioning on $R_{i}\in {ℛ}_{j}$. The magnitude and direction of *δ*_*ij*_ will in turn depend on the selection model.

It is possible to derive an approximation for *δ*_*ij*_ under certain conditions. If *p*(*T*_*i*_|*X*_*i*_, *v*_*i*_) is the standard fixed‐ or random effects meta‐regression model in Equation (2), and $p\left(R_{i}\in {ℛ}_{j}|T_{i},X_{i},v_{i}\right)$ follows the log‐linear model in (12), and the *f*_*mj*_ are differentiable with respect to *T*_*i*_, then

(13) $$\delta_{\mathrm{ij}}\approx H_{j}\left(X_{i}\beta ,X_{i},v_{i}\right)\left(\tau^{2}+v_{i}\right)\sum_{m=0}^{m_{j}}\psi_{\mathrm{mj}}f_{{\mathrm{mj}}^{\prime }}\left(X_{i}\beta ,X_{i},v_{i}\right),$$

where *H*_*j*_(*X*_*i*_ *β*, *X*_*i*_, *v*_*i*_) is equivalent to pRi∈ℛjTiXivi evaluated at *T*_*i*_ = *X*_*i*_ *β* and

$$f_{{\mathrm{mj}}^{\prime }}\left(X_{i}\beta ,X_{i},v_{i}\right)={\left.\frac{\partial f_{\mathrm{mj}}}{\partial T_{i}}\right|}_{T_{i}=X_{i}\beta }$$

is the derivative of *f*_*mj*_ with respect to *T*_*i*_ evaluated at *T*_*i*_ = *X*_*i*_ *β*. A more detailed proof is presented in Appendix.

While the following sections will examine possible values that *δ*_*ij*_ may take under different selection models, we can gain some insight on bias by examining (13). The expression for *δ*_*ij*_ depends on three main quantities. First, *δ*_*ij*_ is an increasing of *H*_*j*_(*X*_*i*_ *β*, *X*_*i*_, *v*_*i*_), which is the probability that Ri∈ℛj. This implies that the bias will be greater as the probability of omitting an observation increases. Second, *δ*_*ij*_ increases in the sum of variance components *τ* ^2^ + *v*_*i*_, which means that the bias will be larger when *T*_*i*_ varies more around the regression line. Finally, *δ*_*ij*_ depends on *ψ*_*mj*_ *f*_*mj*_^*'*^(*X*_*i*_ *β*, *X*_*i*_, *v*_*i*_). Since *f*_*mj*_^*'*^ is the derivative of *f*_*mj*_ with respect to *T*, when *f*_*mj*_ does not depend on *T*, then *f*_*mj*_^*'*^ = 0, and hence *ψ*_*mj*_ *f*_*mj*_^*'*^ = 0. Thus, *δ*_*ij*_ depends on the components of the selection model that are functions of *T*_*i*_ and how strongly those components are related to the probability of observing *X*_*i*_ via the parameter *ψ*.

## 5 BIAS IN COMPLETE‐CASE ANALYSES

Complete‐case analyses only include effects for which all relevant covariates are observed. The complete‐case coefficient estimator ${\hat{\beta }}_{C}$ given in Equation (7) conditions on *R*_*i*_ = 𝟙. As noted above, conditioning on *R*_*i*_ can induce bias, however there are conditions under which the CCA will lead to unbiased coefficient estimates. These conditions largely amount to whether or not *R*_*i*_ is independent of the effect size estimate *T*_*i*_, the outcome of meta‐regression model. When the distribution of *R*_*i*_ depends on *T*_*i*_, then complete‐case estimators will be biased.

The general condition under which CCA estimators are unbiased is that *R*_*i*_ ⊥ *T*_*i*_, which occurs for different types of selection models. First, if the covariates are MCAR, then *R*_*i*_ ⊥ (*T*_*i*_, *X*_*i*_, *v*_*i*_). Alternatively, if the selection model depends only on *v*_*i*_, but not *X*_*i*_ or *T*_*i*_, then *R*_*i*_ ⊥ (*T*_*i*_, *X*_*i*_)|*v*_*i*_; this would constitute a MAR mechanism. Finally, if the selection model depends only on *v*_*i*_ and *X*_*i*_, but not *T*_*i*_, then *R*_*i*_ ⊥ *T*_*i*_|(*X*_*i*_, *v*_*i*_), which would correspond to an MNAR mechanism. Under each of these assumptions, it can be shown that the model that conditions on complete cases *R*_*i*_ = 𝟙 is identical to the complete‐data model, and hence CCA estimators will be unbiased:

(14) $$p\left(T_{i}|X_{i},v_{i},R_{i}=1,\eta ,\psi \right)=\frac{p\left(R_{i}=1|T_{i},X_{i},v_{i},\psi \right)p\left(T_{i}|X_{i},v_{i},\eta \right)}{p\left(R_{i}=1|X_{i},v_{i},\eta ,\psi \right)}=p\left(T_{i}|X_{i},v_{i},\eta \right).$$

This result is consistent with prior work regarding linear regression models with missing covariates.^35^, ^36^

An important aspect of this result is that whether or not a CCA produces unbiased coefficient estimates depends more on the role of *T*_*i*_ in the selection model rather than traditional mechanism classifications of MCAR, MAR, or MNAR. However, various selection models satisfy the conditions of MAR, and similarly with MNAR, the key factor for bias in CCA estimators is the relationship between *R*_*i*_ and *T*_*i*_. Should *R*_*i*_⊥̸*T*_*i*_, then CCA estimators can be biased, regardless of whether the mechanism is MAR or MNAR. Similarly, if *R*_*i*_ ⊥ *T*_*i*_, CCA estimators can be unbiased, regardless of MAR or MNAR.

When *R*_*i*_ is not independent of *T*_*i*_ (given *X*_*i*_ or *v*_*i*_), then CCA can be biased. Let ${ℛ}_{1}=\left\{1\right\}$ so that the CCA conditions on $R_{i}\in {ℛ}_{1}$. Based on Equation (11), the bias of ${\hat{\beta }}_{C}$ will depend on the *δ*_*i*1_. If we let Δ = [*δ*_11_, …, *δ*_*k*1_] be the vector of *δ*_*i*1_ and let Δ_*C*_ be the subset of Δ for which all covariates are observed (i.e., *R*_*i*_ = 𝟙). Then the bias of the complete‐case analysis can be written as

(15) $$\text{Bias}\left[{\hat{\beta }}_{C}\right]={\left(X_{C}^{T}W_{C}X_{C}\right)}^{-1}X_{C}^{T}W_{C}\Delta_{C}.$$

The bias in Equation (15) is a weighted average of individual biases *δ*_*i*1_. Hence, the bias will be larger if the *δ*_*i*1_ are larger (and in the same direction).

Precisely, how large the bias in (15) is will depend on the distribution of *R*_*i*_ and its relationship to effect estimates *T*_*i*_ and their covariates *X*_*i*_. When *R*_*i*_ follows the log‐linear model in (12), the approximate bias can be written as

(16) $$\text{Bias}\left[{\hat{\beta }}_{C}\right]\approx {\left(X_{C}^{T}W_{C}X_{C}\right)}^{-1}X_{C}^{T}W_{C}H_{1C}f_{1C}\psi_{1},$$

where

$$H_{1}=\mathrm{diag}\left[H_{1}\left(X_{i}\beta ,X_{i},v_{i}\right)\right]$$

is a *k* × *k* diagonal matrix where entries refer to the probability that an observation is *not* a complete case,

$$f_{1}=\left[f_{01^{\prime }}\left(X_{i}^{T}\beta ,X_{i},v_{i}\right),\ldots ,f_{m_{1}1^{\prime }}(X_{i}^{T}\beta ,X_{i},v_{i})\right]$$

is a *k* × *m*_1_ matrix of derivatives, and $\psi_{1}={\left[\psi_{01},\ldots ,\psi_{m_{1}1}\right]}^{T}$ is a vector of parameters that index the selection model. Note that the bias in (16) involves **H**_1C_ which contains the rows of **H**_1_ for which *R*_*i*_ = 𝟙; similarly for **f**_1C_.

While (16) provides a general expression for the approximate bias of ${\hat{\beta }}_{C}$, it can be a little difficult to interpret. Loosely, we can see that the bias depends on the probability that covariates are missing, reflected in **H**_1C_, as well as some function of the components of the log‐linear selection model **f**_1C_ *ψ*_1_. To better intuit this bias, we provide a simple example in the following section.

### 5.1 Example: complete‐case analysis with a single binary covariate

Suppose the model of interest includes a single binary covariate *X*_*i*1_ ≡ *X*_*i*_ ∈ {0, 1}, so that the complete data model is

(17) $$T_{i}=\beta_{0}+\beta_{1}X_{i}+u_{i}+e_{i},$$

where *β*_0_ and *β*_1_ are the regression coefficients of interest. Note that *β*_0_ is the average effect when *X*_*i*_ = 0 and *β*_1_ is the contrast in mean effects for when *X*_*i*_ = 1 versus when *X*_*i*_ = 0.

Because *X*_*i*_ is a scalar, so is *R*_*i*_; *R*_*i*_ = 0 indicates that *X*_*i*_ is missing, *R*_*i*_ = 1 indicates that *X*_*i*_ is observed. A CCA would include only effects *i* for which *X*_*i*_ is observed (i.e., *R*_*i*_ = 1). The complete‐case estimator for *β*_0_ is given by a weighted sum of *T*_*i*_ among the effects for which *X*_*i*_ = 0 and *R*_*i*_ = 1:

(18) $${\hat{\beta }}_{0C}=\frac{\sum_{i:X_{i}=0,R_{i}=1}w_{i}T_{i}}{\sum_{i:X_{i}=0,R_{i}=1}w_{i}}.$$

The complete‐case estimator for *β*_1_ is given by the difference between the (weighted) mean effect for *X*_*i*_ = 1 versus *X*_*i*_ = 0:

(19) $${\hat{\beta }}_{1C}=\frac{\sum_{i:X_{i}=1,R_{i}=1}w_{i}T_{i}}{\sum_{i:X_{i}=1,R_{i}=1}w_{i}}-{\hat{\beta }}_{0C}.$$

Assume that the selection model is log‐linear, and that for the sake of simplicity the probability of observing *X*_*i*_ depends on the size of the effect *T*_*i*_ and the value of *X*_*i*_:

(20) $$\text{logit}\left[p\left(R_{i}=1|T_{i},X_{i},v_{i}\right)\right]=\psi_{0}+\psi_{1}T_{i}+\psi_{2}X_{i}.$$

Note that this is an MNAR mechanism, since the probability *X*_*i*_ is observed depends on *X*_*i*_ itself; a MAR mechanism would involve *ψ*_2_ = 0 in Equation (20). Because (20) depends on *T*_*i*_, *δ*_*ij*_ ≠ 0 for this selection model regardless of MAR or MNAR (i.e., regardless of whether *ψ*_2_ = 0 or not), the CCA estimators may be biased.

Under this model, *H*_1_(*X*_*i*_ *β*, *X*_*i*_, *v*_*i*_) depends only on *X*_*i*_ and not *v*_*i*_, so we can write $H_{1}\left(X_{i}\right)=p{\left.\left(R\neq 1|T_{i},X_{i},v_{i}\right)\right|}_{T_{i}=X_{i}^{T}\beta }$. As well, *f*_11_(*T*_*i*_, *X*_*i*_, *v*_*i*_) = *T*_*i*_ and *f*_21_(*T*_*i*_, *X*_*i*_, *v*_*i*_) = *X*_*i*_. Given the result in Equation (13), we can write

(21) $$\delta_{i1}\approx H_{1}\left(X_{i}\right)\left(v_{i}+\tau^{2}\right)\psi_{1}.$$

Given the selection model in (20), the bias of the complete‐case estimator for the intercept, ${\hat{\beta }}_{0C}$, is:

(22) $$\text{Bias}\left[{\hat{\beta }}_{0C}\right]\approx H_{1}\left(0\right)\left({\bar{v}}_{0}+\tau^{2}\right)\psi_{1},$$

where ${\bar{v}}_{0}$ is the average estimation error variance *v*_*i*_ among effects for which *X*_*i*_ = 0 and *R*_*i*_ = 1. The expression in (22) depends on three key quantities, and is an increasing function of each of those quantities. First, the bias increases in *H*_1_(0), which is an approximation of the probability that *X*_*i*_ is missing among studies for which *X*_*i*_ = 0. While under model (20), this probability is a function of *T*_*i*_ and *X*_*i*_, we can intuit *H*_1_(0) loosely as a missingness rate in *X*_*i*_ among effects for which *X*_*i*_ = 0. Second, the bias in (22) is increasing in ${\bar{v}}_{0}+\tau^{2}$, the average variation of *T*_*i*_ for which *X*_*i*_ = 0; the greater the variation, the greater the bias. Because the *v*_*i*_ is typically decreasing in sample size, if studies have smaller samples, the bias will be greater. Finally, the bias depends on *ψ*_1_, which characterizes the relationship between an *X*_*i*_ being observed (i.e., *R*_*i*_) and *T*_*i*_. When *ψ*_1_ is positive, larger effect estimates *T*_*i*_ are more likely to have observed *X*_*i*_ and the bias will be positive; if *ψ*_1_ is negative, so that larger effect sizes are more likely to be missing the covariate *X*_*i*_, then the bias will be negative.

To gain better insight into Equation (22), suppose $v_{i}\approx v={\bar{v}}_{0}$ so that each study has roughly the same estimation error variance. If we assume *T*_*i*_ is on the scale of a standardized mean difference, *v*_*i*_ ≈ 4/*n*_*i*_ where *n*_*i*_ is the total sample size used to compute *T*_*i*_. Various researchers have described conventions for the magnitude of *τ* ^2^ that range from *τ* ^2^ = *v*/4 to *τ* ^2^ = *v*.^37^, ^38^, ^39^ Thus, we can write *τ* ^2^ + *v* = 4(1 + *r*)/*n* from some constant *r* that ranges from 0 to 1.

Further, the parameter *ψ*_1_ is a log‐odds ratio, which reflects the odds of a complete case for *T*_*i*_ versus *T*_*i*_ − 1. There are various conventions for the size of an odds ratio that depend on base rates *P*[*R* = 𝟙|*T*] that could be interpreted as ranging from 1.5 to as large as 9.0, though various researchers have noted that odds ratios greater than 3.0 or 4.0 could be considered large.^40^, ^41^, ^42^, ^43^ Thus, we consider a range of odds ratios from about 1.5 to 4.5. However, the actual size of *ψ*_1_ will depend on the scale of a change in effect size $D_{T}=|T_{i}-{\tilde{T}}_{i}|$. Since it corresponds to a difference, *D*_*T*_ should be no larger than an individual |*T*_*i*_|. Based on conventions in the social and medical sciences (some arbitrary, some empirical), meaningful values of *D*_T_ might feasibly range from 0.2 to 1.0.^40^, ^44^ These conventions for odds ratios and *D*_T_ would imply that relevant values of |*ψ*_1_| might range from 0.4 (large *D*_T_ with small odds ratio) to over 7.5 (small *D*_T_ with large odds ratio).

**FIGURE 1:**
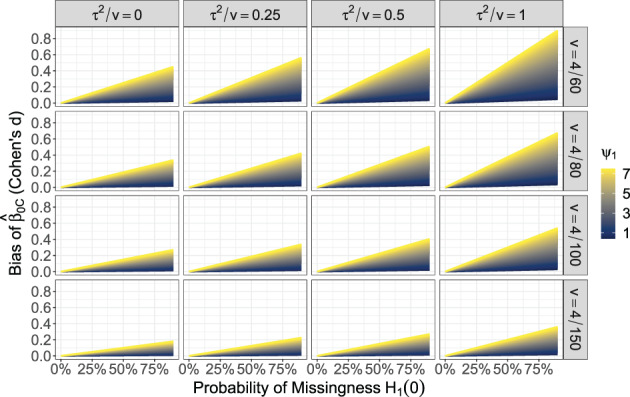
The bias of the intercept estimate ${\hat{\beta }}_{0C}$ (*y*‐axis) of the example. Bias is shown as a function of the average sampling variance *v*, residual heterogeneity *τ* ^2^, the probability of missingness when *X*_1_ = 0, *H*_1_(0) (*x*‐axis), and the correlation between missingness and the effect size as measured by *ψ*_1_ (color). Note that *ψ*_1_ is a log‐odds ratio for effect sizes on the scale of Cohen's *d* [Colour figure can be viewed at wileyonlinelibrary.com]

Based on these conventions, Figure 1 shows the potential (approximate) bias of ${\hat{\beta }}_{0C}$ for this example. Each panel corresponds to a given within‐study variance *v* = 4/*n* and residual heterogeneity *τ* ^2^. Panels plot the bias contributed by a single case *δ*_*i*_ as a function of the probability of missingness *H*_1_(0) (*x*‐axis) and *ψ*_1_ (color). The panels on the bottom few rows and left most columns show that if both *ψ*_1_ is small and *τ* ^2^ + *v* is small, then *δ*_*i*_ will be less than 0.05. However if *τ* ^2^ + *v*_*i*_ is larger and the probability of a complete case is strongly related to *T*_*i*_ (i.e., *ψ*_1_ is large), then the bias can be greater than *d* = 0.2 or even 0.5.

It is worth noting that Figure 1 gives the bias for when *T*_*i*_ is positively correlated with *R*_*i*_, and hence *ψ*_1_ > 0. When *ψ*_1_ < 0, then the bias of ${\hat{\beta }}_{0C}$ is negative, and would be a mirror image of those in Figure 1. Larger, more negative values of *ψ*_1_ would lead to a greater downward bias.

The bias of the slope coefficient, ${\hat{\beta }}_{1C}$, under selection model (20) is given by:

(23) $$\text{Bias}\left[{\hat{\beta }}_{1C}\right]\approx \left[H_{1}\left(1\right)\left({\bar{v}}_{1}+\tau^{2}\right)-H_{1}\left(0\right)\left({\bar{v}}_{0}+\tau^{2}\right)\right]\psi_{1},$$

where ${\bar{v}}_{1}$ is the mean *v*_*i*_ among effects for which *X*_*i*_ = 1 and *R*_*i*_ = 1. As with ${\hat{\beta }}_{0C}$, the bias of ${\hat{\beta }}_{1C}$ is an increasing function of *ψ*_1_. If *T*_*i*_ has a strong positive correlation with *R*_*i*_, then *ψ*_1_ will be larger and so will the bias of ${\hat{\beta }}_{1C}$.

When all studies have approximately the same estimation error variance so that *v*_*i*_ ≈ *v* and ${\bar{v}}_{0}\approx {\bar{v}}_{1}$, then the bias of ${\hat{\beta }}_{1C}$ is approximately:

(24) $$\text{Bias}\left[{\hat{\beta }}_{1C}\right]\approx \left[H_{1}\left(1\right)-H_{1}\left(0\right)\right]\left(v+\tau^{2}\right)\psi_{1}.$$

The expression in (24) is similar to (22), and both expressions depend on similar quantities. Like ${\hat{\beta }}_{0C}$, the bias of ${\hat{\beta }}_{1C}$ is an increasing function of *τ* ^2^ + *v* and *ψ*_1_. The bias of ${\hat{\beta }}_{1C}$ also increases as a function of *H*_1_(1) − *H*_1_(0), which can be thought of as a difference in missingness rates between cases where *X*_*i*_ = 1 and *X*_*i*_ = 0. Note, however, that this does not imply that MAR data necessarily leads to an unbiased slope estimate. Recall that *H*_1_ is an approximation of the probability *X*_*i*_ is missing given *X*_*i*_ and *T*_*i*_ in (20): *P*[*R*_*i*_≠ 𝟙|*T*_*i*_, *X*_*i*_]. Even if *X*_*i*_ were MAR (i.e., assuming *ψ*_1_ ≠ 0 but *ψ*_2_ = 0), the slope estimate would be unbiased only if the slope was zero: *β*_1_ = 0. This is because when *β*_1_ ≠ 0, we would expect different rates of missingness among studies for which *X*_*i*_ = 1 than *X*_*i*_ = 0 because of the relationship between *R*_*i*_ and *T*_*i*_, as well as the relationship between *T*_*i*_ and *X*_*i*_. Viewed this way, the bias of ${\hat{\beta }}_{1C}$ will be greatest when there are fewer complete cases, missingness is strongly related the value of the covariate *X*_*i*_ or to the size of effects (assuming that effects are correlated with *X*_*i*_).

To gain insight into the magnitude of bias in (24), consider the values of *ψ*_1_ ∈ [0.4, 7.5] and *τ* ^2^ + *v* = 4(1 + *r*)/*n* discussed above. Note that the difference *H*_1_(1) − *H*_1_(0) = *p*(*R* = 0|*X* = 1, *η*) − *p*(*R* = 0|*X* = 0, *η*) is a difference in conditional probabilities. For reference, because both *R*_*i*_ and *X*_*i*_ are binary, then *p*(*R* = 0|*X* = 1) − *p*(*R* = 0|*X* = 0) would be equal to the correlation between *R*_*i*_ and *X*_*i*_ (assuming equal marginals in a 2 × 2 table). Thus, |*p*(*R* = 0|*X* = 1) − *p*(*R* = 0|*X* = 0)| could be as small as 0, but could possibly be as large as 1; arbitrary conventions on the size of correlations suggest that |*p*(*R* = 0|*X* = 1) − *p*(*R* = 0|*X* = 0)| = 0.5 would be a “large” value.^40^

**FIGURE 2:**
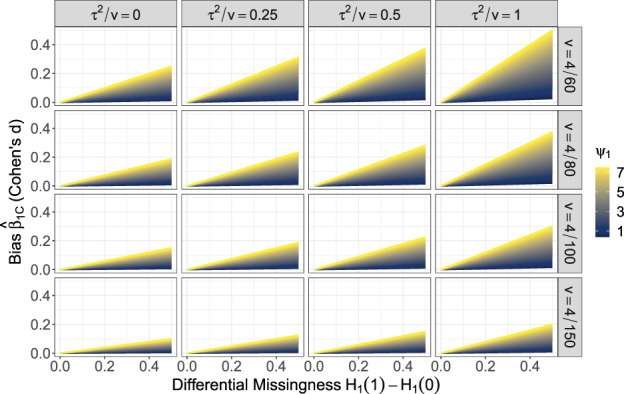
The bias of ${\hat{\beta }}_{1C}$ (*y*‐axis). Each panel corresponds to a given value of residual heterogeneity *τ* ^2^ and estimation error variance *v*. Within panels, the bias of ${\hat{\beta }}_{1C}$ is plotted as function of differential missingness rates (*p*(*R* = 0|*X* = 1) − *p*(*R* = 0|*X* = 0)), which is analogous to the correlation between the value of *X* and whether it is observed. Bias is also shown as a function of *ψ*_1_, which is the relationship between the probability of observing X, and the effect size *T*. Bias is shown on the scale of Cohen's *d* and *ψ*_1_ is on the scale of a log‐odds ratio [Colour figure can be viewed at wileyonlinelibrary.com]

Figure 2 shows the potential bias of ${\hat{\beta }}_{1C}$ for this example assuming the values of *τ* ^2^ + *v*, *ψ*_1_, and *H*_1_(1) − *H*_1_(0) discussed above. Each panel corresponds to a given amount of heterogeneity *τ* ^2^ + *v*, and within panels the bias is shown as a function of the difference *H*_1_(1) − *H*_1_(0) (*x*‐axis) and *ψ*_1_ (color). Figure 2 highlights that the relationship between *R*_*i*_ and *T*_*i*_ (*ψ*_1_) and between *R*_*i*_ and *X*_*i*_ (*x*‐axes) can affect the magnitude of the bias. If *R*_*i*_ is strongly correlated with both *X*_*i*_ and *T*_*i*_ the bias can be as large as *d* = 0.3 or 0.4. However, the less *R*_*i*_ depends on *T*_*i*_ or *X*_*i*_, the lower the bias is.

Recall that the mechanism in these computations is assumed to be MNAR, since *ψ*_2_ in (20) is nonzero. A MAR mechanism would require *ψ*_2_ = 0. In that case, the bias for the CCA intercept estimator ${\hat{\beta }}_{0C}$ is identical to that given in (22). However, the bias in the slope will be slightly different when *ψ*_2_ = 0. This is because, as noted in (24), the bias in the slope depends (loosely) on the correlation between *R* and *X*. Given the form of *H*_1_(*X*) in this example, it is possible for the bias of ${\hat{\beta }}_{1C}$ to be greater when *ψ*_2_ ≠ 0 (MNAR) than when *ψ*_2_ = 0 (MAR), which can occur if the correlation between *R* and *T*, and *R* and *X* are in the same direction (i.e., *ψ*_1_ and *ψ*_2_ are in the same direction). However, when *ψ*_2_ ≠ 0 (MNAR), the bias of ${\hat{\beta }}_{1C}$ can also decrease in magnitude relative to when *ψ*_2_ = 0 if *ψ*_1_ and *ψ*_2_ are in the opposite directions.

A key implication of this example is that under the relatively simple selection model in (20), CCA intercept estimators can have substantial bias. This bias does not change even if *ψ*_2_ = 0 and the data are MAR. Thus, inferences for the group of studies for which *X*_*i*_ = 0 will be biased. Moreover, because inference for the group of studies for which *X*_*i*_ = 1 will depend on the intercept estimate, those inferences will also be biased even if the slope estimator ${\hat{\beta }}_{1C}$ is unbiased.

## 6 BIAS IN SHIFTING‐CASE ANALYSES

Shifting‐case analyses (SCA) are a common approach in meta‐regression when there are very few complete cases across multiple covariates. These analyses involve fitting multiple regression models, where each model omits some of the covariates of interest. In this sense, SCA can be thought of as a set of regression models. Consider one model from that set, which estimates regression coefficients for some subset *S* of the relevant covariates using the estimator ${\hat{\beta }}_{S}$ in Equation (8). Recall that *E* refers to the set of covariates omitted from the model, and that the estimator ${\hat{\beta }}_{S}$ conditions on a set of missingness patterns $R_{i}\in {ℛ}_{j}$. The set of missingness patterns ${ℛ}_{j}$ is such that *R*_*iS*_ = 1 so that all included covariates are observed.

To understand the conditions under which ${\hat{\beta }}_{S}$ is unbiased, we can write a shifting‐case model as:

(25) $$p\left(T_{i}|X_{iS},v_{i},R_{i}\in {ℛ}_{j},\eta ,\psi \right)=\frac{p\left(R_{i}\in {ℛ}_{j}|T_{i},X_{\text{iS}},v_{i},\psi \right)p\left(T_{i}|X_{\text{iS}},v_{i},\eta \right)}{p\left(R_{i}\in {ℛ}_{j}|X_{\text{iS}},v_{i},\eta ,\psi \right)}.$$

The model in (25) is slightly different from the models in the previous sections in that all of the functions depend on the covariates included in a given regression *X*_*i*S_ rather than the complete set of relevant covariates *X*_*i*_. Thus, the function *p*(*T*_*i*_|*X*_*i*S_, *v*_*i*_) can be thought of as a partial‐data model, since it omits some of the relevant covariates. The partial‐data model *p*(*T*_*i*_|*X*_*iS*_, *v*_*i*_) need not be equivalent to the complete‐data model *p*(*T*_*i*_|*X*_*i*_, *v*_*i*_) because the former conditions only on *X*_*iS*_ and not the full set of covariates *X*_*i*_. These models would only be equivalent if *T*_*i*_ ⊥ *X*_*i*E_|*X*_*i*S_, *v*_*i*_. That is, unless the excluded covariates are completely unrelated to effect size (given the covariates included in the SCA model), then ${\hat{\beta }}_{S}$ will be biased even if *X*_*iS*_ are completely observed.

The model in (25) suggests a very strict set of conditions for which ${\hat{\beta }}_{S}$ is unbiased which concern the missingness mechanism and the relevance of excluded covariates in a given shifting‐case regression. First, missingness must be independent of effect sizes. This arises if *R*_*i*_ ⊥ *T*_*i*_|*X*_*i*S_, *v*_*i*_ or *R*_*i*_ ⊥ (*T*_*i*_, *X*_*i*S_)|*v*_*i*_, which is a similar assumption as that made for unbiased CCA. In effect, this assumption implies that missingness is independent of effect sizes *T*_*i*_ (and potentially covariates), but could be correlated with estimation error variances *v*_*i*_.

Second, any excluded covariates must be completely irrelevant to effect sizes given the included covariates: *T*_*i*_ ⊥ *X*_*i*E_|*X*_*i*S_, *v*_*i*_. This assumption is equivalent to assuming that *β*_*j*_ = 0 for all *j* ∈ *E*, so that any omitted variables in a given shifting‐case regression are assumed to have a coefficient of zero. A related assumption is that (*T*_*i*_, *X*_*i*S_) ⊥ *X*_*i*E_|*v*_*i*_, which would imply that the complete‐data likelihood involves no interactions between *X*_*i*S_ and *X*_*i*E_ and that *X*_*i*S_ and *X*_*i*E_ are orthogonal. Given the nature of many meta‐analyses wherein included studies and effects are ostensibly “found objects,” correlation among multiple covariates is a common issue in meta‐regression.^25^ Note that conditions on omitted covariates *and* omitted observations must hold in order for ${\hat{\beta }}_{S}$ to be unbiased.

When the assumptions about omitted variables and effect sizes are not met, ${\hat{\beta }}_{S}$ will be biased. The magnitude of the bias will depend on a number of factors, including the amount of missingness, the missingness mechanism, and the relevance of any excluded covariates. The bias can be expressed as:

(26) $$\text{Bias}\left[{\hat{\beta }}_{S}\right]={\left(X_{\mathrm{US}}^{T}W_{U}X_{\mathrm{US}}\right)}^{-1}X_{\mathrm{US}}^{T}W_{U}X_{\mathrm{UE}}\beta_{E}+{\left(X_{\mathrm{US}}^{T}W_{U}X_{\mathrm{US}}\right)}^{-1}X_{\mathrm{US}}^{T}W_{U}\Delta_{\mathrm{jU}},$$

where **X**_UE_ is the matrix of omitted covariates and *β*_E_ comprises the coefficients for the omitted covariates. The term Δ_*j*_ is a vector of biases due to missingness Δ_*j*_ = [*δ*_1*j*_, …, *δ*_*kj*_] and Δ_*jU*_ is the subset of Δ_*j*_ for which $R_{i}\in {ℛ}_{j}$. Note that the *δ*_*ij*_ are the biases due solely to missingness as in Equation (11).

The expression in (26) shows that a shifting‐case analysis suffers from two sources of bias. The first source, captured in the first term in (26), is a function of the coefficients for the excluded covariates *β*_*E*_, which we refer to as omitted variable bias. Discussion in a previous section argued that the term *omitted variable bias* assumes that *β* is the target of inference in an SCA, which may or may not be the case. If *β* is the target of inference, omitted variable bias arises even if no *X*_*i*S_ are missing, and is related to the issue of multicollinearity in linear models. In fact, if the columns in **X**_US_ and **X**_UE_ are orthogonal, so that the omitted variables are independent of the included variables, then the omitted variable bias will be zero. When the omitted variables are not orthogonal to the included variables, the bias will be nonzero, and it will depend in large part on the contribution of the omitted variables in the complete‐data model **X**_UE_ *β*_E_. The estimator ${\hat{\beta }}_{S}$ will have greater bias if the coefficients for the omitted variables *β*_E_ are larger and the omitted covariates **X**_UE_ are correlated with the included covariates **X**_US_.

The second term in (26) captures the bias due to ignoring observations missing *X*_*i*S_. This missingness bias is a function of Δ_*jU*_, which is itself a vector of biases for each effect, and it can be understood in terms of its individual components *δ*_*ij*_. Because the *δ*_*ij*_ are of the same form for the complete‐case and shifting‐case models, the missing data bias for an SCA is governed by similar factors as the CCA, and are quite possibly similar in magnitude. Based on (13), *δ*_*ij*_ will be positive if *T*_*i*_ is strongly correlated with whether $R_{i}\in {ℛ}_{j}$, and *δ*_*ij*_ will be greater in magnitude when that correlation is larger.

Taken together, shifting‐case estimators can be even more biased than complete‐case estimators. This occurs if the omitted variable and the missingness biases are in the same direction (e.g., both are positive). For both biases to be in the same direction, correlation between *T*_*i*_ and the omitted variables *X*_*i*E_ must be in the same direction as the correlation between *T*_*i*_ the probability that *X*_*iS*_ is observed. If, however, the omitted variable and missingness biases are in opposite directions, this can reduce the bias of a shifting‐case estimator. It is worth noting, however, that it will almost always be impossible to confirm the direction of biases, since they depend on potentially unobserved covariates.

### 6.1 Example: shifting‐cases analysis with two binary covariates

Suppose *X*_*i*_ = [1, *X*_*i*1_, *X*_*i*2_] and *X*_*i*1_ and *X*_*i*2_ are binary covariates such that the regression model of interest is

(27) $$T_{i}=\beta_{0}+\beta_{1}X_{i1}+\beta_{2}X_{i2}+u_{i}+e_{i}.$$

If there is missingness in both *X*_*i*1_ and *X*_*i*2_, then *R*_*i*_ ∈ {0, 1}^2^ so that *R*_*i*_ = [1, 1] indicates both covariates are observed, and *R*_*i*_ = [1, 0] indicates only *X*_*i*1_ is observed. If missingness is such that *R*_*i*_ = [1, 1] for very few effect estimates, then an SCA might involve regressing *T*_*i*_ on the observed values of *X*_*i*1_ and then on the observed values of *X*_*i*2_.

The first regression would take only rows for which *X*_*i*1_ is observed, so that $R\in {ℛ}_{1}=\left\{\left[1,1\right],\left[1,0\right]\right\}$ and the excluded *X*_*i*2_ could be either 0 or 1. The shifting‐case estimators follow from Equation (8):

$${\hat{\beta }}_{0S}=\frac{\sum_{X_{1}=0}w_{i}T_{i}}{\sum_{X_{1}=0}w_{i}},\;{\hat{\beta }}_{1S}=\frac{\sum_{X_{1}=1}w_{i}T_{i}}{\sum_{X_{1}=1}w_{i}}-{\hat{\beta }}_{0S}.$$

Assume that missigness follows the following log‐linear model:

(28) $$\text{logit}\left[p\left(R_{i}\in {ℛ}_{1}|T_{i},X_{i1},v_{i}\right)\right]=\psi_{0}+\psi_{1}T_{i}+\psi_{2}X_{i1}.$$

Note that this gives the log‐odds that an effect is included in the model given *T*_*i*_ and *X*_*i*1_, and that *X*_*i*2_ is not involved. Further, because the distribution of *R*_*i*_ depends on *X*_*i*1_, the mechanism is MNAR.

Given the selection model in (28), the bias of the coefficient estimators can be written as:

(29) $$\text{Bias}\left[{\hat{\beta }}_{0S}\right]=\beta_{2}\frac{\sum_{X_{1}=0,X_{2}=1}w_{i}}{\sum_{X_{i}=0}w_{i}}+\frac{\sum_{X_{i1}=0}w_{i}{\tilde{\delta }}_{i1}}{\sum_{X_{i1}=0}w_{i}},$$

(30) $$\text{Bias}\left[{\hat{\beta }}_{1S}\right]=\beta_{2}\left(\frac{\sum_{X_{i1}=1,X_{i2}=1}w_{i}}{\sum_{X_{i1}=1}w_{i}}-\frac{\sum_{X_{i1}=0,X_{i2}=1}w_{i}}{\sum_{X_{i1}=0}w_{i}}\right)+\left(\frac{\sum_{X_{i1}=1}w_{i}{\tilde{\delta }}_{i1}}{\sum_{X_{i1}=1}w_{i}}-\frac{\sum_{X_{i1}=0}w_{i}{\tilde{\delta }}_{i1}}{\sum_{X_{i1}=0}w_{i}}\right).$$

Here ${\tilde{\delta }}_{i1}$ are the missingness biases as defined above, and whose approximate values is given in (13). To distinguish from the *δ*_*i*1_ from the complete‐case example, we use the $\tilde{\delta }$ notation.

Both the bias of ${\hat{\beta }}_{0S}$ and ${\hat{\beta }}_{1S}$ depend on two terms. The first term in each expression is the omitted variable bias, and the second term in each expression is the missingness bias. Consider the omitted variable biases, which characterize differences between *β*_S_ and *β*. Note that model (27) inherently specifies *β*_0_, *β*_1_, and *β*_2_ as parameters of interest. Because of this, we consider omitted variable bias as relevant to the appraisal of ${\hat{\beta }}_{S}$. When effects are estimated with roughly the same precision, so that *w*_*i*_ ≈ *w*, then the omitted variable biases reduce to

(31) $$\text{Omitted}\;\mathrm{Var}.\;\text{Bias}\left[{\hat{\beta }}_{0S}\right]=\beta_{2}p\left(X_{2}=1|X_{1}=0\right),$$

(32) $$\begin{array}{l} \text{Omitted}\;\mathrm{Var}.\\ \text{Bias}\left[{\hat{\beta }}_{1S}\right]=\beta_{2}\left[p\left(X_{2}=1|X_{1}=1\right)-p(X_{2}=1|X_{1}=0)\right]. \end{array}$$

The omitted variable biases for each coefficient can be seen as depending on two quantities. Both (31) and (32) are increasing in *β*_2_, which is the contribution of *X*_*i*2_ to the complete‐data model. The omitted variable bias for ${\hat{\beta }}_{0S}$ is also increasing in *p*(*X*_2_ = 1|*X*_1_ = 0). The bias for ${\hat{\beta }}_{1S}$ in (32) is increasing in *p*(*X*_2_ = 1|*X*_1_ = 1) − *p*(*X*_2_ = 1|*X*_1_ = 0). Because both *X*_*i*1_ and *X*_*i*2_ are binary, this difference is roughly equivalent to their Pearson correlation (assuming equal marginals). If *X*_*i*1_ ⊥ *X*_*i*2_, then their correlation is zero, and the omitted variable bias will be zero. However, if *X*_*i*1_ and *X*_*i*2_ are correlated, the bias of ${\hat{\beta }}_{1}$ will depend on how strongly correlated *X*_*i*1_ and *X*_*i*2_ are, and how big *β*_2_ is.

**FIGURE 3:**
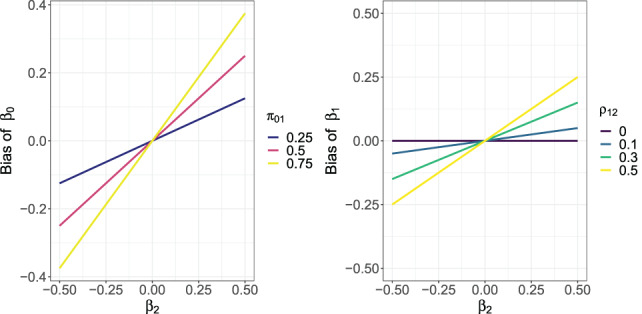
The omitted variable bias of ${\hat{\beta }}_{0S}$ and ${\hat{\beta }}_{1S}$ for model (27) as a function of the omitted variable coefficient *β*_2_. The bias (*y*‐axis) and *β*_2_ (*x*‐axis) are on the scale of Cohen's *d*. The bias displayed is solely due to omitting *X*_*i*2_ from model (27). In the left plot, lines are colored according to *π*_01_ = *p*(*X*_*i*2_ = 1|*X*_*i*1_ = 0). In the right plot, lines are colored according to *ρ*_12_ = *p*(*X*_*i*2_ = 1|*X*_*i*1_ = 1) − *p*(*X*_*i*2_ = 1|*X*_*i*1_ = 0) [Colour figure can be viewed at wileyonlinelibrary.com]

Figure 3 shows the omitted variable bias of ${\hat{\beta }}_{0}$ (left plot) and ${\hat{\beta }}_{1}$ (right plot) as a function of *β*_2_. Both the bias and *β*_2_ are shown on the scale of Cohen's *d*. In the left plot *π*_01_ = *p*(*X*_*i*2_ = 1|*X*_*i*1_ = 0) is the proportion of *X*_*i*2_ = 1 when *X*_*i*1_ = 0. In the right plot, *ρ*_12_ = *p*(*X*_*i*2_ = 1|*X*_*i*1_ = 1) − *p*(*X*_*i*2_ = 1|*X*_*i*1_ = 0), which is roughly the correlation between *X*_*i*1_ and *X*_*i*2_. Note that because *ρ*_12_ can be intuited as (roughly) a Pearson correlation, the values in the figure include 0, 0.1 (i.e., a “small” correlation), 0.3 (medium correlation), and 0.5 (large correlation).^40^

The figure shows that if *β*_2_ = 0 so that *X*_*i*2_ is independent of *T*_*i*_ given *X*_*i*1_, that both ${\hat{\beta }}_{0S}$ and ${\hat{\beta }}_{1S}$ will be unbiased. However, when *β*_2_ is nonzero, both estimators will be biased. If *X*_*i*1_ and *X*_*i*2_ are highly correlated, or if *X*_*i*2_ = 1 when *X*_*i*1_ = 0 with high probability, the bias of both estimators will about as large as a “small” effect (i.e., *d* = 0.2) when *β*_2_ is larger than 0.2. For ${\hat{\beta }}_{1S}$ the bias will be less than about *d* = 0.05 when |*β*_2_| ≤ 0.1 or if *ρ*_12_ < 0.5.

Figure 3 does not take into account any bias induced by missingness. However, because the missingness mechanism in (28) is the same as the mechanism for the complete‐case example (20), the missingness bias for ${\hat{\beta }}_{0S}$ is the same as that for ${\hat{\beta }}_{0C}$ in (22), which is shown in Figure 1. Likewise, the missingness bias for ${\hat{\beta }}_{1S}$ is the same as that for ${\hat{\beta }}_{1C}$ in (23), which is shown in Figure 2.

Thus, the total bias of ${\hat{\beta }}_{0S}$ will be the sum of the omitted variable biases shown in Figure 3 and the missingness biases shown in Figures 1 and 2. If both the omitted and missingness biases are on the higher end, the total bias of ${\hat{\beta }}_{0}$ might be as large as *d* = 0.6 to over 1.0. Likewise, the total bias of ${\hat{\beta }}_{1S}$ will be the sum of the omitted variable biases shown in Figure 2 and the missingness biases shown in Figure 3, and can be larger than *d* = 0.6.

As noted above, the missingness bias and omitted variable bias can be in the different directions. For instance, if *β*_2_ < 0 but ${\tilde{\delta }}_{\mathrm{ij}}>0$, then the omitted variable bias for ${\hat{\beta }}_{0S}$ will be negative, but the missingness bias will be positive. In such cases, the bias of the shifting‐case estimators could be smaller than the bias of the complete‐case estimators. However, because the biases depend on unknown (and potentially unobserved) quantities, it will often be impossible to empirically verify the magnitude or direction of the bias.

## 7 IMPLICATIONS FOR EMPIRICAL EXAMPLE

The theoretical results above suggest that there are conditions under which the coefficient estimates from the CCA and SCA of the substance abuse data in Table 2 are substantially biased. However, it will be difficult, if not impossible, to determine just how biased those estimates are, even given the simplified examples in the previous sections. First, the missingness mechanism is not known for the substance abuse data. Even if we assume that the mechanism follows a log‐linear model like that in (20) or (28), the resulting formulas for the bias depend on quantities, such as *ψ* and *η* that are not known, and cannot be estimated in the presence of missing data without further assumptions.

However, one approach to examining bias in the estimates presented in Table 2 would involve stochastically imputing the missing *X*_*ij*_ in the data. In the same vein as multiple imputation (MI), each set of imputed values constitutes a “complete” dataset from which we can compute the parameters relevant to bias.^13^, ^45^ Given an imputed dataset, we can compute (a) the difference in the resulting ${\hat{\beta }}^{\left(i\right)}$ for the *i*th imputed dataset and ${\hat{\beta }}_{S}$, (b) the quantities that govern bias in the formulas above, including *ψ*, *H*(*X*), and *τ* ^2^. This allows us not only to assess the bias, but also to examine which aspects of the missing data are driving it.

As with MI, the accuracy of the resulting estimated quantities depends on the validity of assumptions regarding missingness and the accuracy of the imputation model. Thus, we would urge interpretation of the following results as *potential* biases in the CCA and SCA estimators presented earlier in this article, rather than a precise estimate of the bias. We generated *m* = 1000 imputations using the mice software in the R programming language.^46^ Estimates of *η* were computed using metaphor, specifying a Paule‐Mandel estimator for the variance component *τ* ^2^.^47^ To estimate the log‐linear model selection parameters *ψ* in (28), as well as *H*(*X*), we used a logistic regression with the missingness indicator *R*_*ij*_ and *T*_*i*_ and *X*_*ij*_ as the predictors.

Here, we focus on results for *β*_0_ and *β*_1_. Consider the regression of *T*_*i*_ on *X*_*i*1_ reported in Table 2. We can view this as a single regression in an SCA that includes only observations for which *X*_*i*1_ is observed. As noted above, the resulting estimators of the intercept *β*_0_ and slope *β*_1_ will exhibit bias due to missingness given in (22) and (23) and bias due to omitting variables as in (31) and (32). Recall that the bias due to missingness in an SCA under this model will be similar to the bias derived for a CCA.

**FIGURE 4:**
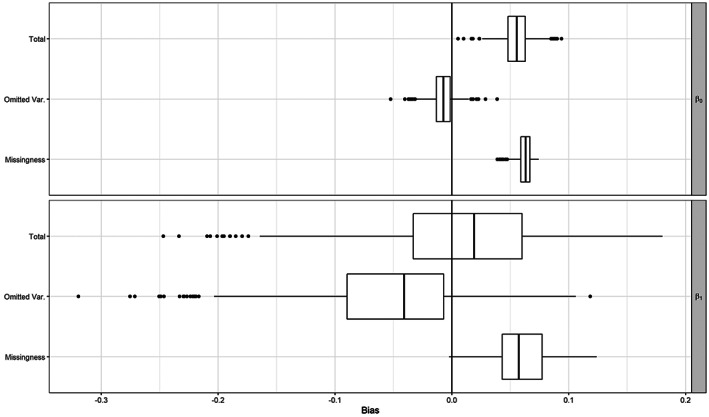
Bias in shifting‐case analysis (SCA) regression of *T*_*i*_ on *X*_*i*1_. For both the intercept *β*_0_ and slope *β*_1_, these boxplots show the total potential bias of the SCA estimators, as well as the omitted variable and missingness bias. Units are shown on the scale of Cohen's *d*

Figure 4 plots the omitted variable bias, missingness bias, and total bias for both ${\hat{\beta }}_{0S}$ and ${\hat{\beta }}_{1S}$. Results are reported on the scale of Cohen's *d*. Omitted variable bias for ${\hat{\beta }}_{0S}$ ranges from −0.05 to 0.04 with a mean of −0.01; omitted variable bias for ${\hat{\beta }}_{1S}$ ranges from −0.32 to 0.12 with a mean of −0.05. Similarly the bias due to missingness could feasibly range from 0.04 to 0.07 with a mean of 0.06 for ${\hat{\beta }}_{0S}$, while the missingness bias of ${\hat{\beta }}_{1S}$ might range from 0 to 0.12 with a mean of 0.06. Note that while the omitted variable bias and missingness bias are in opposite directions in this example, this need not be the case in general; both biases could feasibly be in the same direction for other data. In sum, this amounts to a total bias of 0.01 to 0.09 for ${\hat{\beta }}_{0S}$ and from −0.25 to 0.18 for ${\hat{\beta }}_{1S}$.

## 8 DISCUSSION

This article described a selection model approach to study the bias of two common methods for conducting meta‐regressions with missing covariates: complete‐case and shifting‐case analyses. Under certain assumptions regarding the selection model, we obtained expressions for the approximate bias of coefficient estimators. These expressions were presented in a general form, which was then unpacked by way of examples.

We found that both CCA and SCA will produce biased coefficient estimates unless certain conditions are met. While discussion regarding potential bias of these analyses has largely focused on traditional mechanism taxonomy of MCAR, MAR, and MNAR, we found that bias depends more on the precise model for missingness rather than these broader classifications. Certain mechanisms that are MAR or MNAR can lead to unbiased estimates with CCA and SCA, while other MAR or MNAR mechanisms can induce substantial bias. Complete‐case estimators are unbiased if the probability that all relevant covariates are observed is (conditionally) independent of the effect size estimate. Shifting‐case estimators are unbiased if, in addition to effect sizes being independent of missingness, the covariates omitted from a model have no relationship with the effect size (assuming the full model involving *β* is of interest). When these conditions are not met, the bias of coefficient estimates can be substantial—as large as *d* = 0.4 to *d* = 0.8—depending on the missingness mechanism (i.e., parameters in the selection model), the missingness rate, and the relevance of any omitted covariates.

Results for both CCA and SCA suggest that bias due to missingness will tend to increase in magnitude as a function of the total variation in the data. This means that if studies have small sample sizes (i.e., *v*_*i*_ are large) or there is substantial residual between‐effect heterogeneity *τ* ^2^, the bias of a CCA or SCA will be greater. Because meta‐regression is used to explain between‐effect variation *τ* ^2^, models capable of explaining much of that variation will have lower bias in CCA and SCA estimates. However, even very modest amounts of residual variation can still result in substantial bias.

An important aspect of these findings is that bias will depend on unknown parameters and unobserved data. This means that it will be impossible to empirically verify the magnitude or direction of the bias. Even the estimated biases from the substance abuse data, which were on the order of about *d* = ±0.1 may not be entirely accurate, as so much of that data is missing. Further, it will require strong assumptions regarding the missingness mechanism to correct any bias. These assumptions may be buttressed by theory about scientific reporting, data collection, and data curation.

In addition, it is not immediately clear how commonly the conditions required for unbiased complete‐ and shifting‐case estimators arise. Recent empirical work on examining missingness in meta‐analytic datasets found that effect sizes can be strongly correlated with missingness, though this is not always the case.^48^ Further, the issues of multicollinearity and confounding in meta‐regression, including those discussed by Lipsey,^25^ would suggest that omitting variables in an SCA are likely to induce bias.

Based on these results, our primary recommendation is that analysts attempt to understand the missingness mechanisms and patterns in their data. This can leverage knowledge about standard reporting and coding practices, as well as exploratory analyses.^48^ If there is very little missingness, or if there is a good reason to assume that missingness is uncorrelated with effect size estimates, a CCA may be a reasonable option. However, we would discourage analysts from continuing to use SCA because it would seem unlikely that omitted variable biases are zero in practice.

We would also suggest analysts investigate the feasibility of alternative estimation methods. Ibrahim^32^ describes an EM algorithm for generalized linear models with missing covariates, and Ibrahim, lipsitz, and Chen^33^ extend that algorithm when covariates are MNAR. In addition, full‐information maximum likelihood (FIML) has long been used in linear models,^14^, ^49^ and has shown some promise for meta‐regression involving continuous covariates. Finally, multiple imputation has become something of a standard approach for handling missing data across a number of fields.^13^, ^30^, ^45^

However, employing any of these alternative strategies is not necessarily straightforward for meta‐analysts. To our knowledge, the EM algorithm for missing covariates has yet to be implemented in standard meta‐analytic software. Although FIML for meta‐regression model is available in SEM framework,^50^ the approach has not been empirically validated under various conditions. How best to specify quality imputation models for MI analyses is something of an open question for meta‐regression, as is the potential inaccuracies incurred by using poor imputation models. Research on and clear implementation of these methods for meta‐regression model would seem to be of great use for meta‐analysts.

## CONFLICT OF INTEREST

The authors declare that there is no conflict of interest for this article.

## AUTHOR CONTRIBUTIONS

Jacob M. Schauer, Jihyun Lee, Karina Diaz, and Therese D. Pigott all contributed to the mathematical results and applied examples in this paper.

## Data Availability

The data that support the findings of this study are available from the corresponding author upon reasonable request. Code used in this project is available in a GitHub repository (link: https://github.com/j3schaue/meta_analysis_md_diagnostics/), which contains analysis code (link: https://github.com/j3schaue/meta_analysis_md_diagnostics/blob/master/writeup/cca_paper/properties_of_cca_sca.Rmd). Note that the key findings of this article concern statistical properties of various incomplete data estimators, while the data were used for demonstration purposes only.

## APPENDIX A Approximate bias for log‐linear selection models

### A.1

**Proposition:** Suppose *p*(*T*_*i*_|*X*_*i*_, *v*_*i*_) is the standard fixed‐ or a random effect meta‐regression model in Equation (2), and suppose pRi∈ℛjTiXivi=HjTiXivi follows the log‐linear model in (12). Then:

(A1)
$$E\left[T_{i}|X_{i},v_{i},R_{i}\in {ℛ}_{j}\right]\approx X_{i}\beta +H_{j}\left(X_{i}\beta ,X_{i},v_{i}\right)\left(\tau^{2}+v_{i}\right)\sum_{m=0}^{m_{j}}\psi_{\mathrm{mj}}f_{{\mathrm{mj}}^{\prime }}\left(X_{i}\beta ,X_{i},v_{i}\right),$$

where $f_{{\mathrm{mj}}^{\prime }}\left(X_{i}\beta ,X_{i},v_{i}\right)=\frac{\partial }{\partial T_{i}}f_{\mathrm{mj}}{\left.\left(T_{i},X_{i},v_{i}\right)\right|}_{T_{i}=X_{i}^{T}\beta }$. Therefore, the bias of the conditional expectation is given by:

(A2)
$$\delta_{\mathrm{ij}}\approx H_{j}\left(X_{i}\beta ,X_{i},v_{i}\right)\left(\tau^{2}+v_{i}\right)\sum_{m=0}^{m_{j}}\psi_{\mathrm{mj}}f_{{\mathrm{mj}}^{\prime }}\left(X_{i}\beta ,X_{i},v_{i}\right).$$

**Proof:**

In this proof, we drop the subscript *i* for sake of simplicity. Denote

HjXβ,X,v≡HjX,v=PR=∈ℛj∣T,X,vT=XβGjX,v=1−HjX,vgjX,v=PR∈ℛj∣X,v.

Then an approximation for $E\left[T|X,v,R\in {ℛ}_{j}\right]$ is as follows:

$$\begin{array}{l} \text{omitting �subscripts},�\text{Taylor series for exponents in}\;P\left[R\in {ℛ}_{j}|T,X,v\right]\\ \;\mathrm{at}\;T=\mathrm{X\beta}\\ \begin{array}{rcl} E\left[T|X,v,R\in {ℛ}_{j}\right] & = & \int \frac{T\exp\left\{-\frac{{\left(T-\mathrm{X\beta}\right)}^{2}}{2\left(\tau^{2}+v\right)}+\sum_{i}\psi_{\mathrm{ij}}f_{\mathrm{ij}}\left(T,X,v\right)\right\}}{g_{j}\left(X,v\right)\sqrt{2\pi \left(\tau^{2}+v\right)}\left(1+e^{\sum_{i}\psi_{\mathrm{ij}}f_{\mathrm{ij}}\left(T,X,v\right)}\right)}\mathrm{dT}\\ & = & \int \frac{T}{g_{j}\left(X,v\right)\sqrt{2\pi \left(\tau^{2}+v\right)}}\exp\{-\frac{{\left(T-\mathrm{X\beta}\right)}^{2}}{2\left(\tau^{2}+v\right)}\\ & & +\;\sum_{i}\psi_{\mathrm{ij}}f_{\mathrm{ij}}\left(\mathrm{X\beta},X,v\right)+\sum_{i}\psi_{\mathrm{ij}}f_{{\mathrm{ij}}^{\prime }}\left(\mathrm{X\beta},X,v\right)\left(T-\mathrm{X\beta}\right)\\ & & -\log\left(1+e^{\sum_{i}\psi_{\mathrm{ij}}f_{\mathrm{ij}}\left(\mathrm{X\beta},X,v\right)}\right)\\ & & -\;G_{j}\left(X,v\right)\left(\sum_{i}\psi_{\mathrm{ij}}f_{{\mathrm{ij}}^{\prime }}\left(\mathrm{X\beta},X,v\right)\right)\left(T-\mathrm{X\beta}\right)+O\left(T^{2}\right)\}\mathrm{dT}\\ & \approx & \int \frac{T}{g_{j}\left(X,v\right)\sqrt{2\pi \left(\tau^{2}+v\right)}}\exp\{-\frac{1}{2\left(\tau^{2}+v\right)}(T^{2}-2\mathrm{TX\beta}\\ & & -\;2T\left(\tau^{2}+v\right)\sum_{i}\psi_{\mathrm{ij}}f_{{\mathrm{ij}}^{\prime }}\left(\mathrm{X\beta},X,v\right)\\ & & +\;2T\left(\tau^{2}+v\right)G_{j}\left(X,v\right)\left(\sum_{i\in D}\psi_{\mathrm{ij}}f_{{\mathrm{ij}}^{\prime }}\left(\mathrm{X\beta},X,v\right)\right)+\ldots )\}\mathrm{dT}\\ & = & \mathrm{X\beta}+H_{j}\left(X,v\right)\left(\tau^{2}+v\right)\sum_{i}\psi_{\mathrm{ij}}f_{{\mathrm{ij}}^{\prime }}\left(\mathrm{X\beta},X,v\right)■ \end{array} \end{array}$$

Note that this uses a first order Taylor expansion of the log‐linear model at *T* = *Xβ*, and thus assumes the *f*_*mj*_ are differentiable. The approximation will be more accurate if *τ* ^2^ + *v*_*i*_ are small. A more accurate approximation is possible if the *f*_*mj*_ are linear in *T*_*i*_. In that case, only an approximation of the denominator of the log‐linear model is required.
